# An Acoustic Sensing Gesture Recognition System Design Based on a Hidden Markov Model

**DOI:** 10.3390/s20174803

**Published:** 2020-08-26

**Authors:** Bruna Salles Moreira, Angelo Perkusich, Saulo O. D. Luiz

**Affiliations:** Embedded Systems and Pervasive Computing Laboratory, Electrical Engineering Department, Federal University of Campina Grande, Campina Grande, Paraíba 58429-900, Brazil; perkusic@embedded.ufcg.edu.br (A.P.); saulo@dee.ufcg.edu.br (S.O.D.L.)

**Keywords:** gesture recognition, acoustic-based input, artificial neural network, Hidden Markov models

## Abstract

Many human activities are tactile. Recognizing how a person touches an object or a surface surrounding them is an active area of research and it has generated keen interest within the interactive surface community. In this paper, we compare two machine learning techniques, namely Artificial Neural Network (ANN) and Hidden Markov Models (HMM), as they are some of the most common techniques with low computational cost used to classify an acoustic-based input. We employ a small and low-cost hardware design composed of a microphone, a stethoscope, a conditioning circuit, and a microcontroller. Together with an appropriate surface, we integrated these components into a passive gesture recognition input system for experimental evaluation. To perform the evaluation, we acquire the signals using a small microphone and send it through the microcontroller to MATLAB’s toolboxes to implement and evaluate the ANN and HMM models. We also present the hardware and software implementation and discuss the advantages and limitations of these techniques in gesture recognition while using a simple alphabet of three geometrical figures: circle, square, and triangle. The results validate the robustness of the HMM technique that achieved a success rate of 90%, with a shorter training time than the ANN.

## 1. Introduction

Day-to-day activities are a combination of tactile and auditory sensations. We play, listen, and interact with a variety of objects every day. Some of these objects are recording our activities, such as the touch screens of smartphones or tablets. Thus, adding sensory perception to objects and surfaces that surround us daily is an active area of research, and it has generated keen interest within the interactive surface community [1,2,3,4].

By connecting a microphone to a surface, a signal is acquired by an acoustic sensor, and it is then possible to analyze the acquired signal to identify information for the development of tangible acoustic interfaces [5]. These interfaces work based on the principle that, when an object is touched and manipulated, the vibrations it produces can be acquired and analyzed to infer how interaction with the object occurs [6].

Acoustic sensing has been explored as a powerful tool to retrieve information from a user’s interaction with a surface or object. Some of this information can be used by developers to enhance interfaces with new features, or, most importantly, to improve interaction on current interfaces [6], which can provide more expression to user-defined gestures and expand the interaction input language.

In contrast to camera-based gesture recognition systems, all sensor elements of the acoustic detection technique can be integrated into the surface, and this method does not suffer from lighting and occlusion problems [7].

Researchers have explored several acoustic-based input techniques. These techniques are classified either as passive or active approaches. Passive approaches detect a user’s input, acquiring and analyzing sounds generated by the user’s actions, without the need for an active component, only depending on the properties of the object used (its structure, material, or exclusive properties). Besides, for active approaches, an actuator must be used to interact with a surface, such as a loudspeaker.

Acoustic detection has been the subject of intense research in recent decades. Various algorithms for processing acoustic information have been explored. These include Artificial Neural Networks [8,9], Convolution Neural Networks (CNN) [10], Support Vector Machine (SVM) [11,12], Decision Trees [13], and Hidden Markov Models [14]. These efforts led to the development of systems used in various acoustic tasks, including gesture and textures recognition, the spatial location of the surface touch, and object classification.

In the context of this work, our focus is to answer the following Research Questions (*RQ*):

- *RQ1:* What are the tools, applications, and trends involving these machine learning classifiers?
- *RQ2:* What are the signal characteristics when using a machine learning technique to recognize acoustic patterns?
- *RQ3:* What is the most suitable machine learning technique for acoustic gesture recognition applications?

We distinguish from previous work by implementing acoustic-based input in gesture recognition applications comparing two different techniques: Artificial Neural Network (ANN) and Hidden Markov Models (HMM). First, we present a low-cost hardware design that is composed of a microphone, a stethoscope, a conditioning circuit, and a microcontroller. Subsequently, we integrated these components with a surface to implement a passive gesture recognition system. Second, we evaluated the dataset acquired for developing and training the ANN and HMM models. For ANNs, we investigate algorithms to process the signal to achieve higher accuracy rates due to the low success rate obtained using unprocessed signals. Finally, we evaluated the HNN model. The results validate the robustness of the HMM technique that achieved a success rate of 90%, with a shorter training time than the ANN. We also discuss how to generate code from the trained HMM, cross-compile it automatically, and embed it into low-cost hardware. Thus, after training, the system would be independent of MATLAB software, which has a higher cost.

The rest of the paper is organized, as follows. In Section 2, we introduce a review of the related works, while in Section 3 we present the materials and methods. In Section 4, we present the experiments and, in Section 5, the discussion of the results against the defined research questions. Finally, in Section 6 we present the conclusions and future work.

## 2. Acoustic Sensing Related Works

In this section, we present a review of scientific papers that use the acoustic detection technique, focusing on the three research questions defined in the introduction.

Nine keywords were defined in order to search for papers aiming to answer the research question enumerated in the introduction, and are as follows:

*Acoustic Sensing, Scratch Input, Acoustic Tracking, Tactile Input, Gesture Classification, Sound based classification, Sound Sensing, Acoustic-based input, and Fingertip Gesture.*

Besides, we defined two general selection criteria: (i) the papers were peer-reviewed and (ii) English was the language used to write the paper.

Scratch Input [13] is an acoustic input technique that is based on the sound originated when a nail is dragged over the surface of textured material. Harrison and Hudson [13] used a microphone attached to a modified stethoscope, particularly suitable for amplifying sound and detecting high-frequency noise. A microphone converts the sound into an electrical signal, which is then amplified and acquired by a computer using an audio input connector. This hardware can be effortlessly attached to existing surfaces, turning them into large, non-powered, ad hoc entry surfaces.

The acoustic pattern recognizer proposed by Harrison and Hudson [13] employs a decision tree based on peak count and amplitude variation. A more sophisticated recognition mechanism can incorporate other dimensions, e.g., frequency and duration, and it may support a broader set of gestures and higher precision. Subsequently, other works started to use more advanced techniques, such as Hambone [14] with HMM, Stane [8] with ANN, and TapSense [12] with SVM.

Hambone [14] is a light and discreet system that offers quick access and the ability to multitask for the interaction of mobile devices. Hambone [14] uses two small piezoelectric sensors placed on the wrist or ankle of the user. When a user moves either the hands or feet, the sounds generated voyage to the device via bone conduction. Subsequently, it transmits the signals digitally to a mobile device or computer for processing using HMMs. Afterwards, the signals are mapped to a set of commands that control applications.

Two limitations were found in the current implementation of Hambone [14]. Whenever a user makes a gesture, there is a two-second lag from the initiation of the gesture to have the final command issued and, when training the system to recognize more than four gestures, the HMM training sessions become time-consuming.

Stane [8] is a small device with an embedded microphone and several textures projected onto its surface. The device classifies the sounds produced when rubbing different areas. Stane [8] uses a two-stage classification process, with instantaneous low-level classification and higher-level classifiers that aggregate the evidence of the first stage over time. For instant classification, the input audio is classified by Multi-Layer Perceptron (MLP). However, Stane [8] needs a textured device case and it cannot be used on any surface, differently of what we are proposing in this paper: a passive acoustic-based input classification easily incorporated into a surface.

TapSense [12] uses a microphone connected to an interactive surface to differentiate between touches of different objects and parts of the finger, and it uses the SVM classifier. Nevertheless, the following amounts of data points were collected per participant to train this classifier: 200 data points for the mobile input set, 160 data points for the table finger set, and 280 data points for the table toolset. It has been identified that the user boredom and fatigue became problematic in more extended study durations.

Most recently, we can find papers using machine learning techniques with high computational costs, such as a combination of motion and audio features to train the neural network, such as Pentelligence [9], or even using Convolutional Neural Network, such as Mingshi et al. [10]. Indeed, it is required massive amounts of data, millions of samples.

For example, Pentelligence [9] is based on a pen for handwritten digit recognition. It can be used on regular paper, and no separate dedicated tracking device is required. The authors of Pentelligence [9] found that combining the strengths of audio and motion features produces better results when motion data are classified first. Neural networks trained on the sound emissions of the pen tip are used for evaluation purposes. However, the authors of Pentelligence [9] have used a contact sensor, a microphone, an accelerometer, and a gyroscope in order to achieve a reasonable recognition rate. All of these sensors and the high computational cost became problematic in low-cost applications.

Chen et al. [10] explore the possibility of extending the input and interactions beyond the screen of the mobile device by using ad hoc adjacent surfaces. The proposed system Ipanel uses the acoustic signals from the fingers’ sliding on the table for tracking. Different features are extracted by exploiting the spatio-temporal and frequency domain properties of the produced acoustic signals. The features are transformed into images, and then Chen et al. [10] employed CNN to recognize the finger movement on the table. Ipanel can support not only commonly used gestures (such as click, flip, scroll, and zoom) recognition and handwriting (10 numbers and 26 letters) recognition at high accuracy.

Further, Ipanel’s performance is robust against different levels of ambient noise and different surface materials. Although, the recognition accuracy significantly decreases when the fingers are 20 cm away from the mobile, mainly because the signals become too weak to be used for recognition, and the five-layer CNN adopted has a high computational cost.

## 3. Materials and Methods

We conducted an experiment focusing on the dataset input for the classifiers in the gesture classification problem to improve the analysis. Our goal is to have simple hardware and low computational cost for the machine learning algorithm that can achieve high success rate with at most ten drawings per gesture and short training time (maximum two minutes per gesture), wherefore the user is not fatigued in the training stage. Such a goal is similar to the one that motivated the authors of TapSense [12].

Based on a previous study, several papers focus on gesture recognition using a subset of machine learning techniques, but some of the most common techniques with low computational cost are ANN and HMM. Therefore, the next section presents our platform, analyzes these two machine learning techniques, and compares them.

### Platform

**Figure 1:**
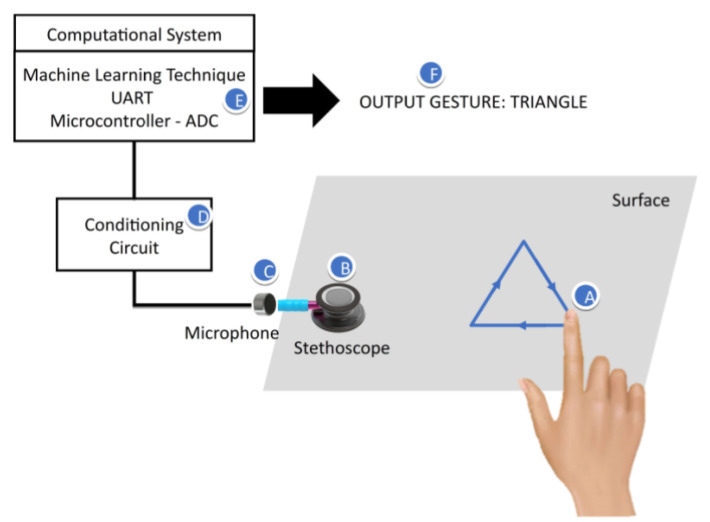
Functioning of the proposed system.

Figure 1 illustrates the operation of the proposed system. When a nail (A) scratches a surface, it produces a series of mechanical vibrations, which propagate through the surface and they are acquired by the microphone (C), which is connected to the stethoscope cannula (B). The use of stethoscopes to acquire sounds naturally provides a high level of suppression of environmental noise. The microphone converts the sound into an electrical signal. By covering the microphone with the stethoscope, the first may pick up a less noisy signal from the environment. This allows for impacts to be promptly segmented from any background noise with a simple amplitude threshold [15]. The microphone signal is then filtered and amplified by a conditioning circuit (D), and acquired by an analog to digital converter of a microcontroller. Then the microcontroller sends the digital signal via serial communication (UART) to the computer (E), which finally will be capable of recognizing the gesture performed (F) on the surface by one of the machine learning applications developed in MATLAB.

The stethoscope has a dual lumen design. The mini electret microphone dimensions are 0.16 × 0.06 inches, and it has to be small enough to fit inside the stethoscope cannula. The conditioning circuit is a low-cost microphone amplifier by Adafruit MAX4466. This circuit has a trimmer pot to adjust the gain from 25× to 125×. The output is rail-to-rail and the supply voltage operation is 2.4 V to 5.5 V.

The microcontroller used in this project is the Arduino Nano V3.0, which has, as primary characteristics, its small size and low cost, allowing greater flexibility for the use of this board in projects whose size is essential. The ADC of this microcontroller is a 10-bit, and the UART transmission rate used in this project is 115,200 bits/s.

Because sound travels through solid and liquid materials much more efficiently than through the air, the surface’s importance is reaffirmed. Thus, while the friction of the nail on a surface only produces an audible and smooth noise, the signal is propagated considerably better through the solid material. This superior sound propagation means that a signal is propagated over a longer distance, but it is also better-preserved [13]. As the speed of sound propagation in aluminum is among the highest among the common materials, around 6300 m/s, an aluminum surface was chosen to carry out the experiments. The stethoscope provides a high level of suppression of environmental noise to the microphone. Still, all of the experiments were performed against the maximum level of ambient noise of 45 dB.

This work aims to compare the robustness of the HMM and the ANN techniques to recognize gestures on surfaces while using a low-cost acoustic sensing gesture recognition system concerning time and physical memory usage. Accordingly, we established the following requirements: ten drawings per gesture at the most, and short training time, a maximum of two minutes per gesture. We defined, for the initial alphabet, three geometrical figures: circle, square, and triangle. Such a small initial alphabet thus prevents time-consuming training. Hence, at the beginning of the application, the user is notified to start drawing one of those figures.

In contrast, for training the recognition of four parts of the finger (tip, pad, nail, and knuckle), Tapsense [12] requires collecting per participant 200 data points for the mobile input set, 160 data points for the table finger set, and 280 data points for the table toolset. When training Hambone [14] to recognize more than 3–4 gestures, the HMM training sessions become extremely tedious. The authors of Tapsense and Hambone found that user boredom and fatigue became problematic in a longer study duration. Furthermore, an excessive number of gestures in the dictionary may lead to high computational costs.

## 4. Experiments

This section describes experiments in order to evaluate the performance of the proposed methods and analyzes them using the machine learning techniques ANN and HMM.

### 4.1. Artificial Neural Networks Experiment

Finding an optimal setup for neural networks is challenging because many parameters have to be considered. Determining the optimal number of hidden layers and neurons for all classifiers is a crucial aspect. If the network’s complexity is too low to model the dataset’s characteristics, the error rates are higher. If the complexity is too high, extended training and recall times are the consequence [16].

Hence, to train the ANN, we collected ten drawings of each gesture (circle, square, and triangle). Subsequently, all of the data were concatenated to be used as input for MATLAB’s Neural Net Pattern Recognition Toolbox. The configuration offered by this toolbox is a two-layer feed-forward network with sigmoid hidden and softmax output neurons. The network is trained with scaled conjugate gradient backpropagation. By setting the number of neurons in the hidden layer to 10 neurons, we had the best performance, because we have minimized the cross-entropy while constraining overfitting. If more than ten neutrons were placed in the hidden layer, the model started to overfit the data and decrease the estimation accuracy.

#### 4.1.1. Dataset for ANN

Achieving a high generalization in one neural network is challenging due to the differences that were observed in our dataset’s scratching styles. The complexity of the optimal topology is possibly too high to be sufficiently trained, given our limited set of training samples. For that reason, we had to process the signal that was presented to train the neural network.

#### 4.1.2. Raw Time Signal

**Figure 2:**
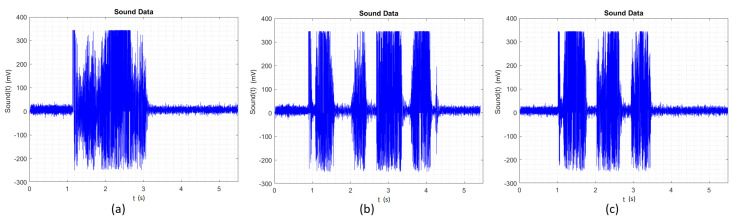
(**a**) Time signal for Circle. (**b**) Time signal for Square. (**c**) Time signal for Triangle.

The first dataset we have used to train the ANN was the raw time signal, as shown in Figure 2. We can see the difference between the square (Figure 2b) and the triangle (Figure 2c) due to the difference in the number of peaks related to the stops at the edges of the drawing. However for the ANN, all of these peaks representing the gesture in the surface (Figure 2) make the ANN classification more complex, because each gesture will have a completely different number and position of peaks and grooves.

Another complicating factor for the neural network is that each person can start the gesture at a different time, making it difficult for the ANN to identify this first peak and classify the signal correctly because each gesture possibly starts at a different time instant. Accordingly, with this dataset, we only had 33% of success.

#### 4.1.3. FFT Signal

**Figure 3:**
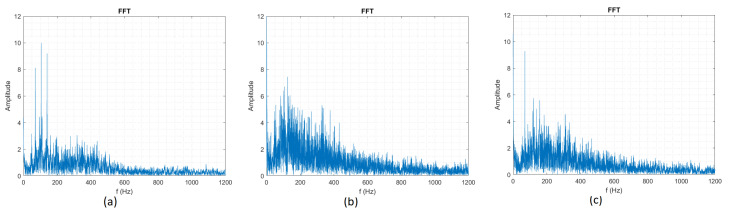
(**a**) Fast Fourier Transform (FFT) signal for Circle. (**b**) FFT signal for Square. (**c**) FFT signal for Triangle.

The second dataset attempt to train the ANN was using the Fast Fourier Transform (FFT) signal, as shown in Figure 3, but it was even harder for the ANN to make the recognition and we had a success rate of 15%.

#### 4.1.4. Envelope Time Signal

**Figure 4:**
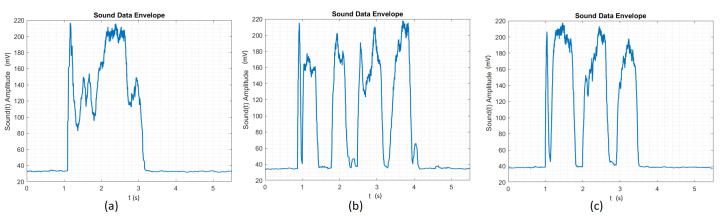
(**a**) Envelope time signal for the Circle. (**b**) Envelope time signal for the Square. (**c**) Envelope time signal for the Triangle.

We have decided to process the raw time signal, as the FFT signal’s performance was worse than that of the raw time signal in the ANN training set. We have used the envelope MATLAB function that returns the upper and lower root-mean-square envelopes of the time signal. The envelope is determined using a sliding window of 200 samples, defined by the author. A sliding window greater than 200 samples results in a signal not generalized enough for the ANN. A sliding window smaller than 200 samples results in a signal which does not preserve the raw signal’s original shape. As shown in Figure 4, the output of the envelope function is greater than or equal to zero, because it returns the root-mean-square envelope of the time signal. The 200 samples sliding window smooths the peaks and preserves the signal shape. Accordingly, it will be more general for the ANN to recognize the gesture, but we still have the problem of when the gesture started to be drawn. For this dataset, we had a success rate of 65%.

#### 4.1.5. Time Scaled Envelope Signal

**Figure 5:**
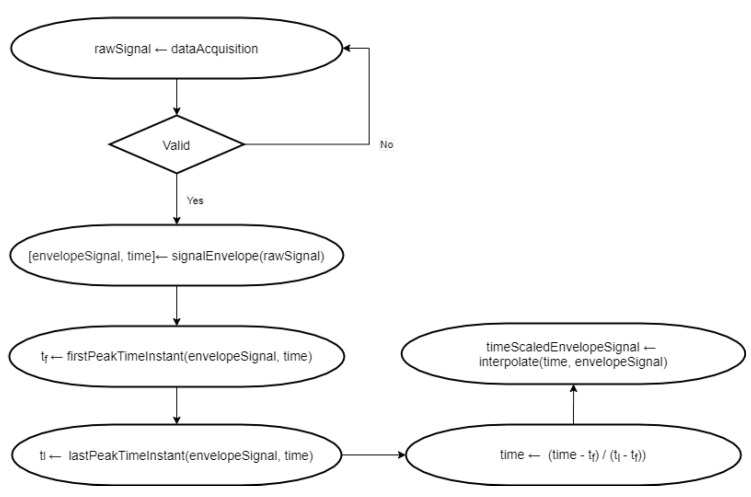
Proposed algorithm for processing the signal.

Finally, we have time scaled the envelope signal employing the algorithm developed in this work and presented in Figure 5. In this data set, we first identify the initial touch, when considering that there is a peak every time the finger touches the surface, and we shift this data set to time 0 (zero). We identify the last touch, and we time scale the data set to the time interval [0, 1], with 100 samples in between. Thus, the instant that the user begins to draw is no longer relevant. The second improvement was making the algorithm robust to the drawing speed, because users have different rhythms. We used time scaling to maintain the time scale of the data across different datasets.

**Figure 6:**
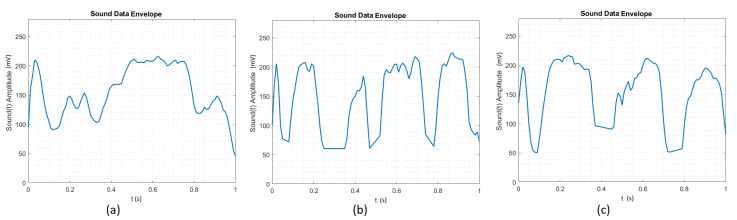
(**a**) Time scaled envelope signal for Circle. (**b**) Time scaled envelope signal for Square. (**c**) Time scaled envelope signal for Triangle.

Furthermore, all of the data sets were the same length of 100 samples. With the find peaks MATLAB function, and the options MinPeakHeight and MinPeakDistance, we could find peaks that had a minimum amplitude and ignored peaks that were very close to each other. Therefore, we have smoothed the peaks and preserved the initial shape of the signal. All of these improvements made it much easier for the ANN recognition task with the enhanced signals shown in Figure 6. Hence, for this dataset, we had the highest accuracy of 90% of success.

Table 1 presents the rate of success of each dataset evaluated to train the neural network. As explained before, the best result was with the time-scaled envelope signal, making it easier for the ANN recognition task.

**Figure 7:**
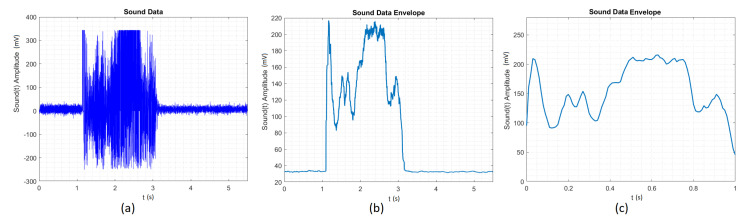
(**a**) Time signal for Circle. (**b**) Time envelope signal for Circle. (**c**) Time scaled envelope signal for Circle.

From Figure 7, one can observe the development of the signal processing for the circle, from the raw signal in time until the time-scaled envelope signal that was evaluated by the algorithm proposed by the authors, as explained in Figure 5. We can see the improvements, as in Figure 7a, we have many more samples (more than 15,000) in the drawing, more peaks, and grooves, and the signal is not general enough for the ANN to have a high (above 85%) recognition rate. In Figure 7b, we have a cleaner signal, with fewer samples and peaks, although we still cannot be general to realize when the drawing has started or the speed of the drawing. Finally, in Figure 7c, we observe the time-scaled envelope signal with only 100 samples. The proposed algorithm smooths the peaks and preserves the initial shape of the signal.

### 4.2. Hidden Markov Models Experiment

A stochastic approach for modeling an acoustic-based input is the Hidden Markov Model technique. A first-order Markov model is a finite set of states, where transitions between states are modeled by a transition probability matrix *A*, while assuming that the probability of being in state $S_{i}$ at time t only depends on the state occupied at time t−1. If the state probability vector π is known for t=0, the probability vector for the next observation moments can be recursively computed by [17]:

(1) $$\pi_{t}=A\times \pi_{t-1}$$

(2) $$\pi_{t}=A_{t}^{0}$$

The state sequence of a Hidden Markov Model cannot be observed directly (hidden), and only the observation sequence is known. A Probability Density Function describes the probability of $P_{i}$ for the observation vector, given that the process is in a given state [17].

Over the past years, hidden Markov modeling has been one of the most popular and effective modeling techniques for acoustic time series. The HMMs are suitable for the classification from one or two-dimensional signals and it can be used when the information is incomplete or uncertain [18]. The reasons why this method has become so popular are the inherent (mathematically accurate) statistical structure; the facility and availability of training algorithms to estimate model parameters from finite sets of speech data training; the flexibility of the resulting recognition system in which you can easily change the size, type, or model architecture to suit words and sounds [19].

Taking into account that the acoustic speech signal has characteristics that are similar to the acoustic signal produced when a nail is dragged over a surface, previous works on speech recognition using HMMs were used as a basis and inspiration for this experiment. It is worth mentioning that the acoustic signal structure is similar, but the complexity of the signal dragged by a nail as compared to the speech signal is lower, since it is neither necessary to search for a range of phonemes nor to have a large database to train the model.

The choice of a topological configuration and the number of states in the model generally reflect the prior knowledge of the specific sound source to be modeled and is not related to mathematical treatability or implementation considerations [19]. The ideal number of states is best determined by executing experiments, because the relationship between the number of states and the HMM performance is very imprecise.

Luigi Rosa [20] proposed a fast and reliable algorithm for speech recognition based on Hidden Markov Models. The proposed approach uses frequency-related features robust to most types of noise.

The topological structure of the HMM implemented is left-right. These settings were pre-chosen by the toolbox of Luigi Rosa and his team [20] because they present good results in recognizing gestures on surfaces. We choose the left-right topology, because the gesture starts and ends in well-identified moments, and a sequential HMM well represents the sequential behavior of the movement.

We developed an application in MATLAB to support gesture data collection and hidden Markov model training. For each gesture, the application prompts the user to start drawing, and then records the microphone signal for a three-second interval and saves it as a .wav extension by the audio write MATLAB function. Once recording completes, the system asks the user whether it should retain the particular drawing. After the application collects five drawings for a particular gesture, it repeats the same data collection process with the next gesture. After collecting data for all three gestures (circle, square, and triangle), the application generates a hidden Markov gesture model via the Luigi Rosa toolbox [20].

**Figure 8:**
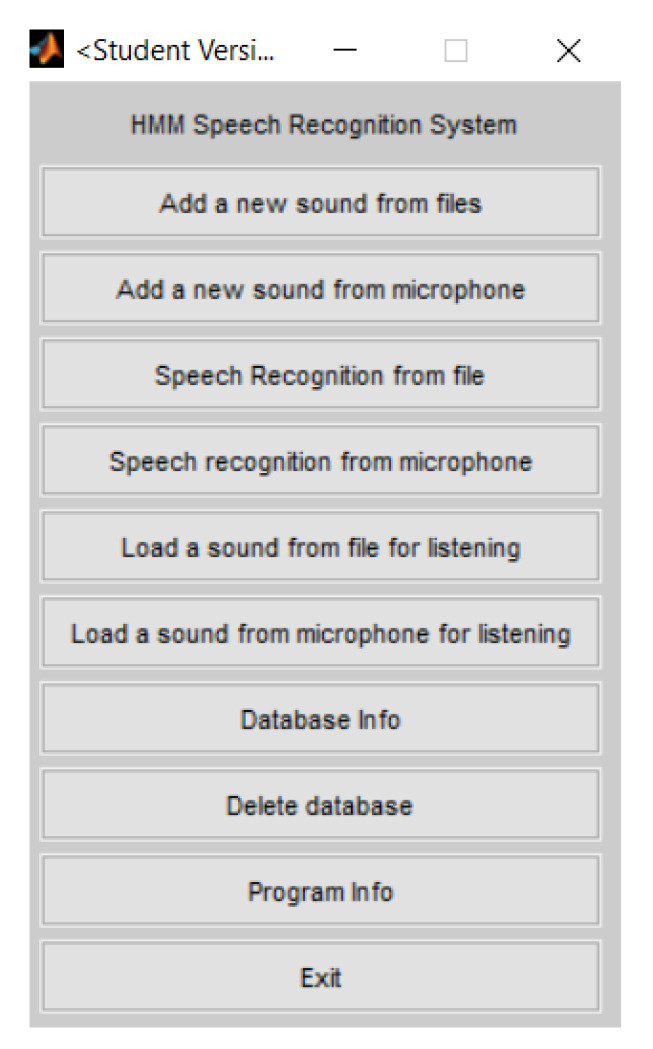
Luigi Rosa [20] interface toolbox in MATLAB.

Figure 8 presents Luigi Rosa interface toolbox in MATLAB. We used the tab *Add a new sound from files* to train the model (it only accepts .wav file extensions).

The first dataset attempt to train the HMM was the raw time signal, such as for the ANN. We added five drawings for each gesture. Subsequently, using the tab *Speech recognition from file* in Luigi Rosa toolbox [20], we loaded a new sample to be recognized, and we obtained a recognition rate of 90%.

## 5. Discussion

We address the research questions and the limitations of our proposed platform in this section when considering the literature review and the experimental results presented in this work.

### 5.1. Addressing RQ1

The *RQ1* is widely addressed in the Acoustic sensing Related Works Section. Nevertheless, based on the related works presented, this section considers the tools that this work uses in more detail.

This paper has no intention of developing the toolboxes to train and build the neural network or the Markov model. Our focus is on the hardware and the embedded software development to acquire and process the signal and improve and test the datasets to feed the models. We used MATLAB’s Neural Net Pattern Recognition Toolbox to train the network using scaled conjugate gradient backpropagation. In this toolbox, we can choose the network parameters, such as the number of neurons in the pattern recognition network’s hidden layer, select samples for validation and testing, and it is possible to evaluate the ANN performance while using cross-entropy and confusion matrices. If the network’s performance is not good enough, we can train it again, adjust the network size, or even import a larger data set to improve the network performance.

For the HMM, we have researched and found some toolboxes for speech recognition, such as the Georgia Tech Gesture Toolkit (GT2k) [21] and the gpdsHMM [18]. However, we had some issues in making them work, and we could not find support. Subsequently, we have tried the MATLAB toolbox implemented by the team of Luigi Rosa [20]. Accordingly, after collecting data for all of the gestures, the application generates a hidden Markov gesture model via Speech Recognition using Hidden Markov Models by Luigi Rosa [20]. We exchanged information with the Luigi Rosa team, and it was straightforward to make it work. Nevertheless, differently from the ANN toolbox, we have used, in this one, we can only supply the data to train the model, and we cannot choose any other parameter of the model. Therefore, the ANN toolbox is more configurable, gives the user more freedom to choose the parameters, and provides graphs that make it possible to evaluate the network’s performance.

### 5.2. Addressing RQ2

A central question when developing systems using machine learning is how much data are needed for training before accuracy levels off. In our work, we have chosen the target precision rate of at least 90%, taking into account the precision rates from most of the related works: 89.5% from Scratch input [13], 90–95% from Hambone [14], 95% from Tapsense [12], 78.4% from Pentelligence [9], 75% from Stane [8], and 91.3% from Ipanel [10]. We have tried the raw time signal and then the FFT signal for the ANN. The latter had the worst performance. We have then processed the raw time signal by taking the envelope and made some improvements such as interpolation to make the peaks and grooves smoother. After all these attempts, we have reached a success rate of 90%, using for each gesture a ten drawings dataset, each composed of 100 samples, 1000 samples per gesture. Finally, for the HMM, we have achieved a success rate higher than 90% by training the model with the raw time signal and five drawings of each gesture, each drawing composed of 15,000 samples, which is, 75,000 samples per gesture.

Consequently, the choice of the dataset and the number of drawings for each machine learning technique is essential. For some drawings, the HMM has mistaken the square for the triangle and vice versa, but for the circle, we had a 100% successful recognition. It is essential to point out that HMM with a smaller number of drawings per gesture, shorter time to train, and smaller computational cost, then the ANN achieved the best precision rate.

### 5.3. Addressing RQ3

A previous work that inspired us was the Ipanel [10], because we have the same problems of tracking the acoustic signals that are generated by sliding fingers on a surface, and Ipanel’s reached a robust performance against different levels of ambient noise and varying surface material. However, Ipanel [10] transformed the features into images and then employed the CNN to recognize the finger movement on the table. We have also tried to develop a CNN in this work, with MATLAB Deep Learning Toolbox. However, to train a network from scratch, the architect is required to define the number of layers and filters, along with other tunable parameters. Training an accurate model from scratch also requires massive amounts of data, on the order of millions of samples ([Online] The MathWorks, Inc, “Convolutional Neural Network” Available: Footnotes are not allowed in this journal, we moved it to here, please confirm. https://www.mathworks.com/solutions/deep-learning/convolutional-neural-network.html, accessed 28 December 2019).

Deep neural networks with a higher amount of training data and different topologies can achieve high recognition rates, as well as the impact of different surfaces or background noises [9]. Nevertheless, as we were looking for a simple and low computational cost solution, the CNN approach (or any deep neural network) turned out to be much more laborious, because of the need for this huge dataset and the longer time to train the network.

Similarly, works, like TapSense [12], also inspired us. For the classification, they use the SVM technique provided in the Weka machine learning toolkit. However, as we can see in their works, training this classifier requires the collection of over 160 data points per gesture. Moreover, SVM also has some weakness when applied to tasks with low computational effort requirement, due to the large memory requirement and computation time of this training algorithm [22]. Consequently, the need for thousands of samples to train the model and the high computational effort would not fit our requirement of at most ten drawings per gesture and short training time (maximum of 2 min per gesture). These SVM limitations led us to try other techniques, such as HMM and ANN.

When considering the success rates for both machine learning techniques presented in Section 4, we achieved the highest success rate through the Markov model technique, which indicates that the proposed solution with HMM is simpler to implement and train than the ANN technique, and it is sufficient to solve the problem stated in the research questions.Therefore, HMM with a smaller dataset, shorter time to train, and computational cost lower than ANN achieved the best result.

### 5.4. Discussion in How to Embed the HMM Trained into the Low-Cost Hardware

In this section, we discuss how to generate code from the trained HMM automatically, cross-compile it, and then embed it into low-cost hardware. Thus, after training, the system would be independent of MATLAB software, which has a higher cost.

**Figure 9:**
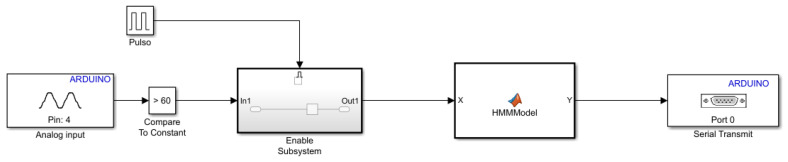
Model developed in Simulink to embedded on the hardware.

The first step is the installation of the hardware support package in Simulink. Second, develop the model, as shown in Figure 9. In this model, the Analog input acquires the raw signal. If the signal value is greater than 60, which means that someone is drawing on the surface, the *Compare To Constant* block applies a True value into the import *In1* of the *Enable Subsystem* block, which has a memory block saving the raw signal during three seconds. The raw signal is then fed into the hidden Markov model previously generated through the MATLAB HMMModel function. Finally, the gesture recognition result (circle, triangle, or square) is printed on the serial output by the *Serial Transmit* block.

It is necessary to load, on the Arduino Nano, 25 MATLAB files, which exceed 140 KB, with a specific input and output relationships, generated by Luigi Rosa’s toolbox [20]. Hence, it is crucial to consider the memory specifications of the microcontroller that the developed HMM is to be embedded.

### 5.5. Limitations

We had some limitations on our platform, as we set our goal to use simple hardware and low computational cost for the machine learning algorithm. First, the maximum level of ambient noise had to be 45 dB. We chose the aluminum surface to carry out the experiments because of its high speed of sound propagation, and the use of stethoscopes to acquire sounds was crucial, because it provides a high level of suppression of environmental noise. Second, we used a simple initial alphabet of geometrical figures: circle, square, and triangle.

We found that small variations in sensor placement caused significant variations in signal characteristics. Thus, repeatable sensor placement is critical for practical inter-session training and recognition.

## 6. Conclusions

In this paper, we presented the design of a low-cost acoustic sensing gesture recognition system based on surface scratch input. In the context of the design, we validated the robustness of the HMM technique to recognizing gestures on surfaces and with low computational costs concerning time and physical memory use. We obtained at least a 90% success rate with five drawings per gesture, and a short training time, a maximum of two minutes per gesture.

Besides the design of the system, we focused on processing the dataset that trains the models to achieve better performance. To do so, we implemented two machine learning techniques, namely ANN and HMM, to support answering the research questions. The solution proposed with the HMM classifier is simpler to implement and train than the ANN technique and it is sufficient to solve the problem. For the training stage, with five drawings per gesture and the raw time signal, the HMM achieved a success rate of 90%. Therefore, the HMM with a smaller number of drawings, shorter signal processing time, and training time obtained the best result. The results also demonstrated the importance of data representation for the efficient processing of sensory information for the ANNs.

In future work, we are investigating the use of multiple microphones for the physical sensing setup providing an extension of using location and improve the classification precision. Additionally, a hardware implementation, e.g., FPGA (Field Programmable Gate Array), can be considered.

## Figures and Tables

**Table 1:** Success rate results for the different datasets that trained the ANN.

Signal	Success Rate
Raw time signal	33.00%
FFT signal	15.00%
Envelope signal	65.00%
Time scaled envelope signal	90.00%
